# A small 7q11.23 microduplication involving *GTF2I* in a family with intellectual disability

**DOI:** 10.1111/cge.13753

**Published:** 2020-04-29

**Authors:** Michele Pinelli, Gaetano Terrone, Flavia Troglio, Gabriella Maria Squeo, Gerarda Cappuccio, Floriana Imperati, Piero Pignataro, Rita Genesio, Lucio Nitch, Ennio Del Giudice, Giuseppe Merla, Giuseppe Testa, Nicola Brunetti‐Pierri

**Affiliations:** ^1^ Department of Translational Medicine Section of Pediatrics, Federico II University Naples Italy; ^2^ Telethon Institute of Genetics and Medicine Naples Italy; ^3^ European Institute of Oncology, IRCCS Milan Italy; ^4^ Division of Medical Genetics Fondazione IRCCS‐Casa Sollievo della Sofferenza San Giovanni Rotondo Italy; ^5^ Department of Molecular Medicine and Medical Biotechnologies University of Naples Federico II Naples Italy; ^6^ Department of Oncology and Hemato‐oncology University of Milan Milan Italy; ^7^ Human Technopole Milan Italy

## Abstract

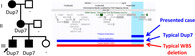

*To the Editor:*

Recurrent deletions and duplications of 7q11.23 region are responsible for Williams‐Beuren Syndrome (WBS) and 7q11.23 microduplication syndrome (Dup7), respectively.^1^ Both 7q11.23 deletions and duplications typically are 1.5 Mb in size and have recurrent breakpoints. However, atypical copy number variants (CNVs) of different sizes and breakpoints affecting the 7q11.23 region have also been reported.^2^ Detailed phenotyping of these atypical cases can inform on the role of genes in the critical region.^2^ For WBS, data from both patients and animal models suggest that *GTF2I* is responsible for the neurodevelopmental phenotype. In contrast, the role of *GTF2I* in the Dup7 is less clear. To date, only one subject presenting with autism spectrum disorder without cognitive impairment and carrying an atypical duplication sparing *GTF2I* has been reported.^3^

We describe an 11‐year‐old male Caucasian proband, first child of non‐consanguineous parents with uneventful medical history until expressive language delay and learning difficulties were noted at 3 and 6 years of age, respectively. No abnormalities were detected by electroencephalography, and ophthalmologic and audiologic evaluations. Brain magnetic resonance imaging showed non‐specific gliosis in the bilateral frontal white matter and a partial *empty sella* without evidence of endocrine dysfunctions. Abdominal ultrasonography showed hepatomegaly with mild steatosis. At 11 years of age, his weight was 76 kg (>95th centile, z‐score = +3.2) and height 144 cm (54th centile) with a body mass index of 36.6 kg/m^2^ (>95th centile, z‐score = +4.6). His head circumference was 56.2 cm (81st centile). Facial features included broad forehead, straight eyebrows, narrow eyelid fissures, deep set eyes, and thin upper lip (Figure 1A). Cognitive impairment and learning difficulties were also present in his younger brother and his mother (Figure 1B). Furthermore, his maternal grandmother had gait imbalance and chronic kidney failure. Both proband's mother and grandmother attended basic school grades and occasionally worked as housekeepers. His younger sister, father and maternal grandfather had no neuro‐developmental problems by report. All three siblings were obese.

**FIGURE 1:**
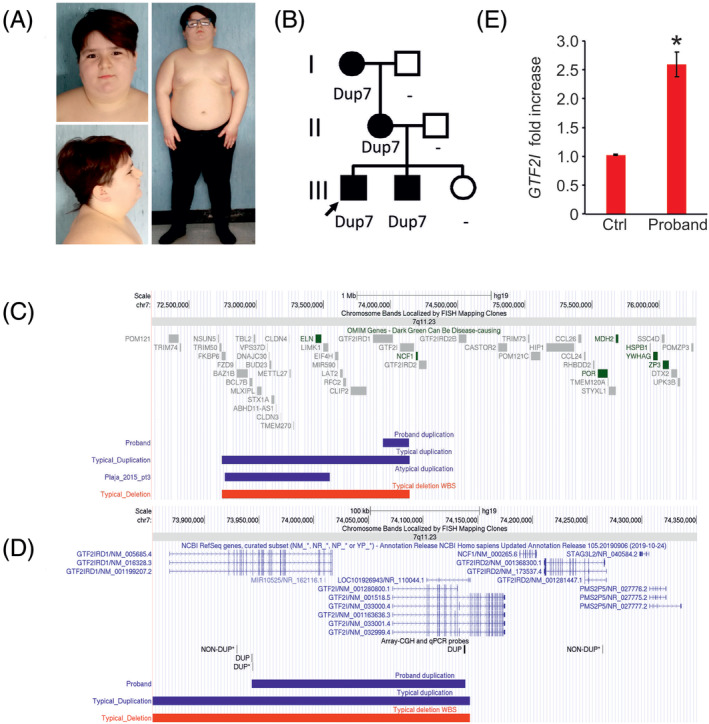
A, Pictures of the proband at the age of 8 years showing broad forehead, straight eyebrows, narrow eyelid fissures, deep set eyes, thin upper lip and obesity. B, Family pedigree. C,D, Snapshot of UCSC genome browser displaying the duplicated region in the proband, in the typical duplications and deletions, and in the case reported by Plaja et al. (patient 3 ‐ pt3).^3^ Both *GTF2IRD1* and *GFT2I* transcripts are on the plus strand and thus, only *GTF2I* Transcription Start Site maps on the duplicated region. The breakpoints of the duplication were mapped by a combination of array CGH and quantitative polymerase chain reaction (qPCR). Data obtained by qPCR are marked with an *. E, *GTF2I* expression on peripheral blood mononuclear cells (PBMC) of the proband and controls. RNA was extracted using RNeasy Mini kit following the manufacturer's directions and quantified by ThermoScientific NanoDrop 8000. A total of 200 ng of RNA was reverse‐transcribed into cDNA using SuperScript VILO cDNA Synthesis Kit (Invitrogen). qRT‐PCR‐TaqMan assays for *GTF2I* and *TBP* (Hs01073660_m1 and Hs00427620_m1) from Applied Biosystems were used to quantify expression in 5 ng cDNA in triplicate. qRT‐PCR TaqMan Fast Advanced Master Mix (Applied Biosystems) was used. Data were normalized to *TBP* and fold changes calculated using the 2^‐ddCt formula using wild‐type PBMC as control. Statistical significance was evaluated by unpaired *t*‐test using Qbase+ software. *P < .01 [Colour figure can be viewed at wileyonlinelibrary.com]

Evaluation of cognitive functioning by Wechsler Intelligence Scale for Children IV (WISC‐IV) or Wechsler Adult Intelligence Scale‐Revised (WAIS‐R) in the proband, his siblings and his mother revealed a mild cognitive impairment with homogenous involvement of functional domains in his brother and mother (Full‐scale IQ of 61, 57, 91 and 62 for the proband, his brother, his sister, and his mother, respectively). In the proband, Vineland Adaptive Behaviour Scales (VABS) showed impaired adaptive behaviour consistent with his overall intellectual functioning and no evidences of psychopathological anomalies at clinical visit or at Child Behaviour Checklist (CBCL 6‐18) testing. Maternal grandmother was unavailable for clinical and neuropsychological evaluations.

After obtaining informed consent, blood samples were collected from the proband, both his siblings, his parents and his maternal grandparents and high‐resolution array‐CGH (PerkinElmer CGX) and genomic real‐time quantitative polymerase chain reaction analyses were performed. A 7q11.23 duplication was detected in the proband, his brother, his mother and his grandmother with a minimum duplicated region spanning between nucleotides 73 944 168 and 74 138 459 (maximum interval between 73 929 917 and 74 264 323) (hg19) that partially overlapped with the typical recurrent Dup7 critical region (Figure 1B,C). The duplication was classified as variant of uncertain significance according to the consensus recommendation of the American College of Medical Genetics and Genomics (ACMG) and the Clinical Genome Resource (ClinGen)^4^ and included the 3′ of *GTF2IRD1*, the intergenic region upstream *GTF2I* and a portion of the 5′ of *GTF2I* (Figure 1D). The telomeric portion of *GTF2I* is embedded into two segmental duplications and thus, the telomeric breakpoint of the duplication could not be precisely determined but it appeared to overlap with the typical Dup7 telomeric breakpoint^5^ and involvement of *NCF1* and *GTF2IRD2* cannot be ruled out.

*GTF2I* expression was found to be elevated in peripheral blood mononuclear cells of the proband compared to controls, consistent with data previously reported in subjects with typical Dup7 (Figure 1E). Consistent with typical Dup7 phenotype,^1^, ^5^ the individuals herein presented also showed mild cognitive impairment with homogenous involvement of functional domains. In conclusion, the familial cases herein reported were found to carry a small 7q11.23 duplication that supports the role of *GTF2I* as critical gene for the cognitive impairment of Dup7.

## DATA AVAILABILITY STATEMENT

Array‐CGH results have been released in DECIPHER (ID 379690) and ClinVar (SCV000998756).
